# Thyroid Eye Disease in the Teprotumumab Era: Pathophysiology, Clinical Activity Assessment, and an Updated Therapeutic Landscape

**DOI:** 10.3390/ijms27209071

**Published:** 2026-10-13

**Authors:** Tara Shooshani, Balaji Cherupalla, Amy Durand, Moe Hein Aung

**Affiliations:** Mitchel and Shannon Wong Eye Institute, Department of Ophthalmology, Dell Medical School, University of Texas at Austin, Austin, TX 78712, USA

**Keywords:** thyroid eye disease, Graves’ orbitopathy, thyroid-associated ophthalmopathy, orbital fibroblast, insulin-like growth factor 1 receptor, teprotumumab, veligrotug, clinical activity score, orbital imaging, quality of life, disease-activity metrics

## Abstract

Thyroid eye disease (TED), the principal extrathyroidal manifestation of Graves’ disease, is an autoimmune orbitopathy. The pathophysiology involves aberrant thyroid-stimulating hormone receptor (TSHR) and insulin-like growth factor 1 receptor (IGF-1R) signaling on orbital fibroblasts, leading to glycosaminoglycan accumulation, adipogenesis, inflammatory edema, and extraocular-muscle enlargement. The active phase is typically self-limited yet medically modifiable, characterized primarily by inflammation, edema, and tissue expansion, whereas chronic inactive disease is dominated by fibrosis and fixed structural change. This US-oriented review aims to provide updates in the field through 2026: (1) summarizing the molecular pathophysiology linking thyroid autoimmunity to orbital remodeling and current drug targets; (2) discussing activity-versus-severity assessment, with appraisal of the Clinical Activity Score (CAS); and (3) explaining the complementary roles of different structural and functional imaging modalities in the evaluation and management of TED. We then follow the therapeutic ladder, from conservative measures through the employment of glucocorticoids and steroid-sparing immunosuppression to the use of targeted biologics. We give particular attention to IGF-1R inhibition, including the use of teprotumumab and veligrotug, and to post-approval results, including otologic and metabolic adverse-event burden, recurrence, treatment sequencing, and the emerging efficacy of the treatment of chronic and low-activity disease. The CAS’s limited inter-observer reliability identifies additional reproducible activity measures as an open need.

## 1. Introduction and Epidemiology

Thyroid eye disease (TED), also termed Graves’ orbitopathy, is the principal extrathyroidal manifestation of Graves’ disease, an autoimmune disorder in which antibodies against the thyroid-stimulating hormone receptor (TSHR) trigger orbital inflammation, extraocular muscle expansion, and orbital adipogenesis [1]. The condition is tightly linked to thyroid autoimmunity: roughly 90% of affected patients have Graves’ hyperthyroidism [2], and only a minority are euthyroid or hypothyroid at presentation [1]. Cardinal features include proptosis, eyelid retraction, restrictive diplopia, periorbital edema, and, in the most severe cases, corneal breakdown or dysthyroid optic neuropathy (DON).

The epidemiology of TED derives largely from population-based work in Olmsted County, Minnesota. The foundational cohort study reported an age-adjusted incidence of 16.0 per 100,000 person-years in women versus 2.9 in men, corresponding to a female-to-male rate ratio of 5.5, with a bimodal age distribution peaking at 40–44 and 60–64 years [3]. A 2024 re-examination reported a contemporary overall incidence of approximately 5 per 100,000 person-years, suggesting a lower current disease burden while preserving female predominance [4]. The pooled prevalence of TED among patients with Graves’ disease is approximately 40% [5]. An analysis of insurance claims of moderate-to-severe disease in the US found a similar adjusted prevalence near 44 per 100,000, with 4:1 female predominance [6].

Most TED is clinically mild—defined as disease with limited impact on daily life and no need for immunosuppression or surgery—with a contemporary distribution of approximately 77% mild, 22% moderate-to-severe, and 1% sight-threatening cases, the latter defined by dysthyroid optic neuropathy or corneal breakdown [1]. Even outside the sight-threatening category, clinically meaningful morbidity is common: restrictive diplopia affects approximately 17% of patients, and proptosis with eyelid retraction can produce exposure keratopathy and, in advanced cases, significant corneal compromise [2]. As such, a small minority warrants clinical urgency. Optic-nerve dysfunction, broadly defined, was present in 6% of the unselected Olmsted cohort [2], whereas frank compressive DON, the principal sight-threatening complication, is commonly cited in roughly 3–5% of cases.

Beyond ocular morbidity, TED carries a heavy quality-of-life and economic burden, with impaired health-state utility, frequent mental-health comorbidity, and substantial sick leave and surgical costs [7,8]. Disease-specific quality of life is impaired across visual-functioning and appearance domains [9]. Cigarette smoking is the dominant modifiable risk factor, with case-control studies reporting increased odds of TED among smokers compared with Graves’ disease controls without orbitopathy and non-thyroid controls, with odds ratios ranging from 1.94–10.1 and 1.22–20.2, respectively [10,11,12].

This US-oriented narrative review aims to update the therapeutic landscape of TED in the era of IGF-1R inhibition, beginning with teprotumumab and extending to newly approved and late-stage agents. Its distinctive emphasis is the interface between molecular mechanism and clinical implementation: CAS reproducibility, imaging as an adjunct to activity assessment, long-term safety and recurrence after IGF-1R treatment, and how US practice differs from that of the European Group on Graves’ Orbitopathy (EUGOGO) and the American Thyroid Association (ATA)/European Thyroid Association (ETA) frameworks.

We performed a targeted narrative review of English-language literature in PubMed/MEDLINE, Google Scholar, ClinicalTrials.gov, and US Food and Drug Administration (FDA) resources, supplemented by a reference-list review of major guidelines and recent reviews. Searches were updated in September 2026, using combined terms including thyroid eye disease, Graves’ orbitopathy, thyroid-associated ophthalmopathy, Clinical Activity Score (CAS), teprotumumab, IGF-1R, veligrotug, IBI311, lonigutamab, linsitinib, batoclimab, tocilizumab, rituximab, orbital imaging, computed tomography (CT), magnetic resonance imaging (MRI), and, in selected settings, single-photon emission computed tomography/computed tomography (SPECT/CT), dysthyroid optic neuropathy, recurrence, retreatment, hearing loss, and hyperglycemia. Peer-reviewed randomized trials, guideline statements, systematic reviews, and large observational cohorts were prioritized. Company press releases and conference/topline reports were used only for rapidly evolving pipeline agents when peer-reviewed publications were unavailable and are identified as preliminary.

## 2. Pathophysiology: What Happens to the Orbit

Thyroid eye disease is an organ-specific autoimmune disorder in which the orbital fibroblast translates systemic thyroid autoimmunity into local remodeling—proptosis, extraocular muscle enlargement, and congestion. Autoantibody-initiated signals are executed as glycosaminoglycan synthesis, de novo adipogenesis, and fibrosis [13]. These heterogeneous cells follow fates set by the receptors they express and the surrounding cytokine milieu (Figure 1).

The trigger for TED is autoimmunity against the thyroid-stimulating hormone receptor (TSHR), the autoantigen of Graves’ hyperthyroidism—hence the clinical link. TSHR antibodies, often reported clinically as thyrotropin receptor antibodies (TRAb), are detectable in more than 95% of patients [1]. The stimulating functional subset—thyroid-stimulating immunoglobulin (TSI)—is particularly relevant to TED because it tracks disease intensity, not merely its presence. Assessed via bioassay, TSI correlated strongly with Graves’ orbitopathy activity and severity, and each patient with diplopia and dysthyroid optic neuropathy was TSI-positive [14]. TSI is higher in active disease and independently predicts steroid response [15], although quantitative TRAb titers also carry similar prognostic weight [16]. Thyroid peroxidase antibodies (TPOAb) may coexist as markers of thyroid autoimmunity, but they are less specific to the orbitopathic mechanism than are TRAb or TSI. K1-70, a human monoclonal TSHR-blocking autoantibody evaluated in early-phase Graves’ disease trials, reduced thyroid-stimulating antibody activity and was associated with proptosis improvement in a subset of patients, supporting the concept that dysfunctional TSHR axis is itself orbitopathic [17].

On the fibroblast, TSHR partners with the insulin-like growth factor-1 receptor (IGF-1R) [18], scaffolded by the beta-arrestin-1 structure such that TSHR engagement potentiates IGF-1R-mediated extracellular signal-regulated kinase (ERK) signaling and hyaluronan synthesis [19]. Whether activation reflects TSHR–antibody crosstalk or direct IGF-1R autoantibodies is debated, as both converge on IGF-1R [13]. TSHR expression rises as fibroblasts mature into adipocytes and correlates with clinical activity, coupling autoimmunity to fat expansion [20,21]. The clinical proof of concept is teprotumumab, an IGF-1R antagonist that produced reduction response in proptosis for 83% of patients versus 10% with placebo [22].

Inflammatory immune signaling, particularly Th17-skewed inflammation and interleukin-6 (IL-6) activity, further amplifies this fibroblast response during active disease. Circulating CD34+ fibrocytes increase IL-6 markedly after TSHR stimulation, linking thyroid autoimmunity to an orbital cytokine milieu [23]. IL-6 promotes B-cell maturation, autoantibody production, and Th17/regulatory-T-cell imbalance, and can signal through both classic and trans pathways via the soluble IL-6 receptor (IL-6R) [24]. The activity of the IL-6R blockade in corticosteroid-resistant disease therefore supports IL-6 as a pathogenic amplifier rather than merely as an inflammatory bystander [25].

Activated fibroblasts remodel the orbit through overlapping adipogenic, edematous, and fibrotic pathways. A subset of fibroblasts undergoes peroxisome proliferator-activated receptor gamma (PPAR-gamma)-mediated adipogenesis, expanding orbital fat and contributing to axial proptosis [21]. Activated fibroblasts and differentiating orbital fibroblast lineages also produce hydrophilic hyaluronan within orbital connective tissue, orbital fat, and extraocular muscles. By drawing water into the orbit, hyaluronan then contributes to both fat expansion and the muscle enlargement, congestion, and edema of active disease [20]. With progression, transforming growth factor beta (TGF-beta)-driven myofibroblast differentiation and collagen deposition produce more fixed fibrosis and restrictive myopathy [13,21]. Together, these pathways explain lipogenic fat-expansion versus myogenic muscle-restrictive phenotypes, while emphasizing that dysthyroid optic neuropathy reflects posterior extraocular-muscle enlargement and apical crowding rather than proptosis alone.

This biology defines the natural history of the disease: an active, inflammatory phase in which fibroblast activation, hyaluronan accumulation, and adipogenesis are at least partly reversible, progressing to a burnt-out fibrotic chronic phase in which collagen deposition renders changes largely irreversible. The trajectory links mechanism to management (Figure 1), i.e., the use of receptor-directed and immunomodulatory therapies during the active window, whereas residual fibrosis and fat expansion require rehabilitative surgery (based on current understanding of the disease and available therapeutic options, which may change in the future).

## 3. Clinical Presentation and Natural History

Thyroid eye disease, the orbital manifestation of Graves’ disease, arises when glycosaminoglycan (GAG) accumulation, adipogenesis, and inflammatory edema expand orbital fat and the extraocular muscles within a closed bony orbit. The hallmark signs follow directly from this volumetric expansion.

Eyelid retraction—upper (Dalrymple sign) and lower—is the most prevalent finding, along with lid lag on downgaze (von Graefe sign), producing the characteristic stare. Proptosis reflects forward globe displacement by expanded retrobulbar tissue. Specifically, interocular asymmetry (>2 mm) and longitudinal change track abnormality more reliably than does any single Hertel threshold, though absolute millimeters govern teprotumumab eligibility and decompression planning. Present in roughly 17% of patients, restrictive myopathy of the fibrotic inferior and medial recti limits excursion causes binocular diplopia [2]. Soft-tissue congestion—periorbital edema, conjunctival injection, chemosis, and caruncular swelling—forms the core of activity scoring.

Ocular-surface disease is near-universal. A widened palpebral fissure, lid lag, reduced blink, and impaired Bell phenomenon expose the cornea to evaporative desiccation and mechanical irritation. This exposure produces punctate epithelial erosions, foreign-body sensation, tearing, photophobia, and exposure keratopathy; in severe cases, persistent epithelial defects, ulceration, or corneal breakdown may develop [26]. EUGOGO guidelines therefore mandate lubrication in all patients, with escalation to gels, ointments, lid taping, moisture chambers, or urgent protective intervention when exposure threatens the cornea [26].

Dysthyroid optic neuropathy (DON) is the other major sight-threatening complication of TED. DON arises chiefly from apical crowding by hypertrophied muscle bellies—not proptosis—and frequently occurs without marked exophthalmos. In the EUGOGO survey, apical crowding was the most consistent finding (95% of eyes) in patients with DON, whereas disc swelling appeared in only 56% of cases. Preserved acuity did not exclude the diagnosis [27]. Male sex, older age, smoking, and diabetes increase the risk of developing DON [28,29]. Because compressive optic atrophy is irreversible, DON is an emergency.

The natural history of TED follows Rundle’s curve, a biphasic trajectory (Figure 2). An active inflammatory phase, typically lasting one to three years, first brings retrobulbar pain, congestion, progressive proptosis, and diplopia. The disease peaks, followed by partial regression to a chronic fibrotic plateau. Critically, activity, not duration, drives treatment. A baseline Clinical Activity Score (CAS) of at least 4 predicted response to immunosuppression with 86% specificity and an 80% positive predictive value [30].

Acute, active disease is medically modifiable, and anti-inflammatory or targeted therapy works best here. Chronic, inactive disease is often dominated by fixed structural changes—residual proptosis, restrictive strabismus, and lid malposition—that are typically managed with staged rehabilitative surgery once activity is quiescent [26]. This distinction, however, is not absolute. Proptosis and muscle restriction may persist after inflammation subsides because they reflect tissue expansion and scarring, but emerging IGF-1R inhibition data suggest that some residual tissue expansion remains biologically modifiable even in long-duration, low-activity disease. Specifically, in a placebo-controlled trial of chronic, low-activity TED, teprotumumab reduced proptosis by 2.41 mm versus 0.92 mm with placebo (*p* = 0.0004), supporting a partially reversible component beyond the classic active inflammatory phase [31].

## 4. Assessment of Disease Activity and Severity

Contemporary management of thyroid eye disease separates disease activity—the reversible inflammatory component that responds to immunomodulation—from disease severity, the cumulative anatomical and functional burden. For more than two decades, disease activity in thyroid eye disease has been assessed primarily using the Clinical Activity Score (CAS), which Mourits and colleagues developed to translate the classic signs of inflammation into a practical clinical scoring system [30].

The initial-visit CAS comprises seven binary signs—spontaneous retrobulbar pain, pain on gaze, eyelid redness, conjunctival injection, eyelid swelling, chemosis, and caruncular swelling—each scored as present (1) or absent (0) (Table 1). At follow-up, three additional change items extend the score to 10 points: increase in proptosis of at least 2 mm, decrease in ocular motility of at least 8 degrees, and decrease in visual acuity of at least 1 Snellen line over 1 to 3 months. Active disease is conventionally defined as a CAS of at least 3 of 7 [32]. The threshold is not arbitrary: in the original cohort, a baseline CAS predicted response to anti-inflammatory treatment with 86% specificity and an 80% positive predictive value, though sensitivity was only 55% [30].

The use of the CAS grading system is simple and rapid; it can be performed at the bedside and was validated against treatment response rather than a structural surrogate. Its prognostic value persists, with a higher baseline CAS independently predicting poor response to intravenous methylprednisolone in moderate-to-severe disease [16].

However, CAS has its limitations. Inter-observer reliability is poor (Krippendorff alpha 0.532). With six experienced observers assessing the same patients, agreement on the total CAS was only moderate, and agreement on the actionable treat-or-not-treat decision was poor; non-lead assessors concurred with the lead ophthalmologist only 64.5% of the time [33]. The soft-tissue signs are weakest, with low agreement on photographic grading [34]; caruncular edema alone shows only slight agreement and an order-related bias, prompting calls for its refinement [35]. Structurally, the binary, equal-item design weights trivial eyelid redness the same as sight-threatening visual loss, producing ceiling effects [32]. Optic neuropathy may emerge at a low CAS, and the score performs poorly in chronic, fibrotic disease, in which it has never been validated.

Because the CAS grades disease activity but not functional severity, two frameworks run in parallel. The European Group on Graves’ Orbitopathy (EUGOGO) stratifies severity into three levels: (1) mild disease that has limited impact on daily life; (2) moderate-to-severe disease that produces clinically meaningful lid, soft-tissue, proptotic, or diplopic morbidity; and (3) sight-threatening disease that is defined by dysthyroid optic neuropathy or corneal breakdown [26]. The VISA classification grades both severity and activity across four domains: Vision, Inflammation, Strabismus, and Appearance [36]. The systems are not interchangeable [32]. Broadly, EUGOGO is the dominant European guideline framework, CAS remains the most widely used activity instrument in trials and clinical practice, and VISA is used primarily as a structured North American clinic-based assessment [37].

Future classification is moving toward reproducible scoring. Photographic and machine-learning systems confirm that the inflammatory signs carry measurable signals [34,38], yet their ceiling, bounded by noisy human labels, underscores rather than resolves the reproducibility problem.

Patient-reported outcomes also play a key role. The disease-specific Graves’ Ophthalmopathy Quality of Life questionnaire (GO-QOL) and brief three-item TED-QOL capture lived burden; the TED-QOL (https://thyroideyedisease.org/category/iteds-resources/ted-qol-survey/ accessed on 28 July 2026) was validated against GO-QOL, with faster completion [39], and a Singapore variant has been described [40]. Such instruments correlate only moderately with objective severity, confirming a persistent gap between measured pathology and lived experience [39].

## 5. Orbital Imaging: CT, MRI, and Selected Functional Imaging

Orbital cross-sectional imaging serves multiple roles in thyroid eye disease: structural delineation of the bony and soft-tissue orbit for surgical planning, assessment of apical crowding for potential DON, and objective quantification of inflammatory activity (e.g., enlargement of extraocular muscles and proptosis) to complement the imperfect CAS, whose live inter-observer reliability is only moderate [33]. Computed tomography (CT) scan and magnetic resonance imaging (MRI) are complementary rather than interchangeable: CT excels at visualization of skeletal structures and is fast and inexpensive, whereas MRI offers superior soft-tissue contrast, avoids ionizing radiation, and uniquely characterizes activity (Table 2) [41].

The cardinal finding in CT is fusiform enlargement of the extraocular muscle (EOM) bellies with tendon sparing (the “Coca-Cola bottle” configuration), favoring the inferior and medial recti, which helps distinguish thyroid eye disease from tendon-involving myositis [41]. Posterior expansion produces apical crowding, the structural substrate for compressive DON and its single strongest independent predictor (adjusted odds ratio 22.1) [42]. CT permits quantitative apex assessment via the Barrett index; a 60% threshold best discriminates DON (79% sensitivity, 72% specificity) [43]. Because CT delineates the lamina papyracea, orbital floor, and deep lateral wall with millimetric detail, it is the modality of choice for planning decompression [41]. Its limitation lies in its inability to grade activity, since muscle enlargement reflects accumulated volume rather than the edema-versus-fibrosis state [41].

In contrast, MRI visualizes the optic nerve and EOMs with higher soft-tissue resolution and without radiation, while quantitative MRI techniques provide complementary measures of tissue composition and inflammatory activity [41,44]. The most established sequence/marker is the fat-suppressed T2 signal-intensity ratio (SIR), which has been found to be significantly higher in active than inactive disease (area under the curve (AUC) 0.852) [45], with later work confirming the inferior-rectus SIR as a discriminator [46]. Quantitative relaxometry adds reproducible values: combining T1 mapping with SIR raised the staging AUC to 0.900 [45]. Furthermore, T2-relaxation mapping has been studied to identify affected extraocular muscles in TED, and its signal were also noted to fall accordingly after immunosuppression, supporting the potential of using it for treatment-response monitoring [47]. Amongst other MRI sequences studied, diffusion-weighted imaging and the apparent diffusion coefficient capture inflamed-tissue water, correlating with CAS [48,49] and flagging DON [42]; dynamic contrast-enhanced perfusion further improves multiparametric staging [50]. Quantitative MRI encompasses signal-intensity measurements, relaxometry, diffusion-based metrics, dynamic contrast-enhanced imaging, and volumetric assessment, with proposed applications in activity staging, detection of optic-nerve involvement, and prediction of treatment response [44]. Collectively, quantitative MRI shows promise for activity staging and treatment-response assessment, although heterogeneity in acquisition and analysis limits standardization for routine clinical use [44]. Moreover, the use of MRI has other practical limitations: it is slower, costlier, and less widely available than CT, and longer scan times may be difficult for symptomatic or acutely ill patients [41].

Therefore, CT imaging is preferred when bony anatomy or surgical mapping governs the decision, such as before decompression and for rapid apex assessment in acute DON [41,43]. On the other hand, MRI should be used when the question regards inflammatory activity, i.e., for distinguishing the treatable edematous phase from burnt-out fibrosis, serial monitoring, optic nerve characterization, or when radiation avoidance is paramount, as in younger patients [41,45,47].

Technetium-99m Diethylenetriaminepentaacetic Acid (99mTc-DTPA) SPECT/CT provides functional information about orbital inflammatory activity by imaging tracer uptake related to blood–tissue barrier permeability and inflammatory involvement of extraocular muscles, lacrimal glands, the optic nerve, and posterior orbital fat. Recent quantitative work reports correlations between uptake metrics, such as mean standardized uptake value (SUVmean) or maximum standardized uptake value (SUVmax), and clinical activity, and a 2026 feasibility study prospectively evaluated multiple quantitative SPECT/CT indicators [51]. However, its current role should be framed as adjunctive and investigational rather than routine: spatial resolution is lower than for either CT or MRI, availability depends on nuclear-medicine infrastructure, and protocols require further standardization and external validation.

## 6. Conservative and Symptomatic Management

Conservative care, as discussed below, is the foundation of management for every patient with thyroid eye disease and is often sufficient on its own in mild disease, which constitutes roughly 77% of presentations [1]. These measures relieve symptoms and may modify the course, but they neither reverse established proptosis nor undo orbital fibrosis. They remain relevant at every severity, layered beneath immunosuppression with glucocorticoids, targeted biologic therapies, or surgical interventions.

Both thyrotoxicosis and hypothyroidism with elevated TSH worsen orbital outcomes, so stable biochemical control is recommended, and rapid swings should be avoided [1]. The route to restore euthyroidism matters: radioactive iodine can worsen orbitopathy through a post-ablative antibody rise, so the EUGOGO and the ATA/ETA advise concurrent glucocorticoid prophylaxis in at-risk patients, favoring antithyroid drugs or thyroidectomy in active disease [26,52].

Smoking cessation is the single most important modifiable intervention. A systematic review found a consistent association with orbitopathy [10], and a contemporary cohort showed current smoking independently predicted disease (adjusted odds ratio 1.76) with a clear dose-response [11]. Smoking also confers treatment resistance and even blunts the response to teprotumumab [53]. The pathogenic mechanism of smoking to TED comprises tissue hypoxia and oxidative stress, which leads to increased fibroblast proliferation, glycosaminoglycan secretion, and adipogenesis [52].

Ocular-surface protection is universal. Guidelines recommend artificial tears at all times, escalating to gels and ointments, with lid taping or moisture-chamber goggles at night, when corneal exposure or lagophthalmos is present [26,37]. The rationale is mechanical: proptosis and lid retraction widen the fissure, exposing the cornea to evaporative desiccation. Lubricants replenish the tear film and dilute inflammatory mediators; gels and goggles physically shield the cornea overnight, preventing exposure keratopathy.

Postural and dietary measures target dependent congestion. Head-of-bed elevation relieves periorbital edema through gravity-dependent venous and lymphatic drainage, though supporting evidence is limited [37]. A low-salt diet is commonly advised on the same grounds as an expert-opinion measure.

Prisms manage diplopia from mild or stable misalignment by optically deviating light to realign the retinal images, restoring single binocular vision in patients whose deviation is either too small for surgery or still changing, making them poor surgical candidates [37].

Selenium has a defined role in mild active disease. In the EUGOGO trial, six-month daily administration of 200 μg sodium selenite improved quality of life, reduced eye involvement, and slowed progression versus placebo, which was sustained at 12 months [54]. The mechanism is antioxidant, as selenoproteins neutralize the reactive oxygen species that drive fibroblast activation. ATA/ETA recommend considering a course for mild active disease, particularly in selenium-insufficient regions, while noting that benefits in selenium-replete populations, such as much of the United States, remain unproven [37].

## 7. Glucocorticoids and Steroid-Sparing Immunosuppression

For moderate-to-severe active thyroid eye disease, intravenous methylprednisolone (IVMP) pulse therapy is the long-standing pharmacologic backbone. The 2021 EUGOGO guideline recommends IVMP plus mycophenolate as the first-line treatment, at an intermediate cumulative dose of 4.5 g over twelve weekly infusions; a higher 7.5 g schedule is reserved for the most severe disease, capped at 8.0 g per cycle [26].

Pulsed intravenous glucocorticoid delivery is favored over daily oral alternatives with regards to efficacy and tolerability. The pivotal head-to-head randomized trial showed a 77% response with IVMP versus a 51% response with oral prednisone (*p* < 0.01), with fewer adverse events observed with intravenous treatment [55]. Mechanistically, glucocorticoids broadly suppress orbital fibroblast activation, T-cell and B-cell cytokine output, and inflammatory edema, inactivating disease in roughly 45–65% of patients [37].

Though the benefit from glucocorticoid treatment is dose-dependent, more side effects are associated with higher doses. In the EUGOGO dose-ranging trial, 12-week improvement was greatest with 7.47 g (52%) versus 4.98 g (35%) and 2.25 g (28%), yet the high-dose advantage was transient, more toxic, and gave no durable benefit (24-week relapse of 21–40% across arms) [56]. A meta-analysis found no significant response difference between the 4.5 g regimen and higher doses [57], and a 2025 systematic review confirmed the medium-dose regimen as the rational default [58].

Intravenous therapy carries a roughly 80% response rate, with therapy-related morbidity of 6.5% and mortality of 0.6%, supporting an 8 g cap [59]. A European survey reported seven fatal events among intravenous users—chiefly acute liver failure, stroke, and pulmonary embolism—with nearly all doses above 8 g cumulative [60]. Therefore, EUGOGO accordingly warns against single doses above 0.75 g, cumulative doses above 8.0 g, and consecutive-day dosing [26].

Durability is the other limitation for steroid therapy, with relapse or deterioration of disease occurred in 31% of TED patients treated with glucocorticoid [37], echoed by a 31.2% reactivation rate after IVMP [61]. These limitations motivate add-ons that consolidate response and prolong remission. Mycophenolate is the best-supported adjunct. In the MINGO trial, the primary week-12 difference was not significant, with separation emerging at later follow-up; combined intravenous methylprednisolone plus mycophenolate produced a higher week-24 response rate than did methylprednisolone alone (71% versus 53%) and a higher sustained week-36 response rate (67% versus 45.5%), without increasing levels of infection or hepatotoxicity when compared with the results for steroid monotherapy [26]. It is therefore recommended by the EUGOGO as a first-line combination therapy for most patients with moderate-to-severe active TED [26]. Other conventional immunosuppressive agents, including cyclosporine, azathioprine, and methotrexate, occupy a second-line role and are generally used as adjuncts to glucocorticoids rather than as a monotherapy. Their use is constrained by agent-specific toxicity, including nephrotoxicity and hypertension with cyclosporine; myelosuppression and hepatotoxicity with azathioprine; and hepatotoxicity, cytopenia, pneumonitis, and teratogenicity with methotrexate [62].

Orbital radiotherapy is also an established adjunct to glucocorticoids in selected patients, particularly for active inflammatory disease with motility restriction, although its role has narrowed in the biologic era. It should be used cautiously in patients with diabetic or hypertensive retinopathy because of concern for radiation retinopathy [26].

## 8. Teprotumumab: Efficacy, Updated Safety, Rebound, and Chronic Disease

Teprotumumab (or teprotumumab-trbw, as assigned by the FDA) is a fully human IgG1 monoclonal antibody against the insulin-like growth factor 1 receptor (IGF-1R). Its January 2020 FDA approval made it the first drug licensed specifically for thyroid eye disease [37]. On the orbital fibroblast, the thyroid-stimulating hormone receptor (TSHR) and IGF-1R form a single signaling complex dependent on IGF-1R activity, so antagonism attenuates the downstream hyaluronan synthesis, adipogenesis, and cytokine production driving orbital expansion [13]; this direct complex has since been corroborated structurally [18]. Teprotumumab thus interrupts the local autoimmune signalosome rather than broadly suppressing immunity.

In the phase 3 OPTIC trial, 83 patients with active, moderate-to-severe TED were randomized to eight infusions of teprotumumab or placebo over 21 weeks [22]. A proptosis response (≥2 mm reduction without fellow-eye worsening) occurred in 83% versus 10% of patients (*p* < 0.001; mean change −2.82 mm versus −0.54 mm), with diplopia improvement in 68% versus 29% [22]. A pre-specified phase 2/OPTIC pooled analysis (*n* = 171) confirmed a responder rate of 77% versus 15% (difference 63%; 95% CI 51–75) [63]. These reductions are of a magnitude previously achievable only surgically (Section 10).

However, clinical initiation should be individualized rather than driven by CAS alone. In US practice, teprotumumab is generally considered for patients with clinically meaningful TED-related proptosis and/or diplopia after confirming the diagnosis, documenting severity and activity, reviewing prior disease course and treatment response, and assessing contraindicating or high-risk comorbidities. Practical prerequisites include stable thyroid management when feasible, baseline Hertel exophthalmometry and motility/diplopia assessment, review of corneal and optic-nerve status, baseline glycemic assessment, baseline audiologic evaluation or otologic history, pregnancy testing and contraception counseling for patients of reproductive potential, and screening for current or prior inflammatory bowel disease [64,65]. Sight-threatening DON or corneal breakdown should be treated as an emergency and should not be delayed for elective biologic planning.

Several reports also suggest that IGF-1R inhibition can reduce intraocular pressure, probably by reducing orbital congestion and pressure-related venous outflow resistance; however, these data are based on small observational studies and case series, and teprotumumab should not be used as a substitute for standard glaucoma therapy [66,67].

Real-world experience with teprotumumab has raised the concern that some adverse effects may be more frequent or persistent than initially appreciated. In the premarketing placebo-controlled trials, muscle spasms occurred in 25% of teprotumumab-treated versus 7% of placebo-treated patients, hyperglycemia in 10% versus 1%, and hearing impairment in 10% versus 0%; the prescribing information also warns that hearing impairment may be permanent [64]. In a multicenter real-world cohort of 131 patients, teprotumumab-related adverse events occurred in 81.7% of patients, with a median of four adverse events per patient; 46% had at least one persistent adverse event at last follow-up, and 12.2% discontinued therapy because of adverse events [65].

Otologic toxicity is the signal of greatest concern. In a prospective cohort of TED patients treated with teprotumumab, 81.5% developed new or worsening otologic symptoms, yet hearing loss resolved in only 45.5% of affected ears [68]. A second study found high-frequency audiometric changes in most patients with paired data [69]. A large Food and Drug Administration Adverse Event Reporting System (FAERS) analysis found a strong disproportionality signal (proportional reporting ratio of 23.6)—markedly over-represented hearing-loss reports, a signal rather than an incidence estimate—most without resolution [70]. In another multicenter cohort, ear and labyrinth adverse events occurred in 30.5% of patients, subjective hearing impairment occurred in 13.7%, and half of those with hearing impairment had persistent symptoms at last follow-up [65].

Hyperglycemia is likewise more common in practice: a real-world study found it in 52% of patients, affecting all baseline diabetics, with the three-month hemoglobin A1c rising by 1.3 percentage points in diabetics and only 36.4% of these cases returning to baseline [71]. Other effects include alopecia, dysgeusia, menstrual disorders, and infusion reactions. The current US prescribing information lists no formal contraindications to teprotumumab, but pregnancy/fetal harm and inflammatory bowel disease (IBD) are key safety issues [64]. Patients of reproductive potential require pregnancy testing and contraception, and patients with current or prior IBD require careful counseling and monitoring; discontinuation should be considered if IBD is suspected [64,65].

Monitoring and counseling protocols have thus evolved since teprotumumab’s introduction (Figure 3). Before treatment, it is highly recommended to obtain baseline audiogram and otologic history, fasting glucose and HbA1c levels, a pregnancy test in women of childbearing potential, and screening for IBD history and gastrointestinal symptoms. During treatment, emphasis is placed on monitoring hearing and interrupting therapy for new symptoms; tracking glucose and intensifying diabetes co-management; maintaining contraception through therapy and about 6 months after the last infusion; and discontinuing treatment if IBD is suspected [64,65]. Structured monitoring is needed to help anticipate and manage these risks [72].

Recent extension studies have clarified the durability of teprotumumab response under trial conditions. In the OPTIC-X extension, 89.2% of placebo-crossover patients became proptosis responders, week-48 proptosis response was maintained in 90.6% of initial responders, and re-treatment was effective in 5 of 8 patients who flared [73]. Integrated long-term follow-up reported that 82% required no additional TED treatment over 99 weeks [74]. Real-world experience in dysthyroid optic neuropathy has suggested a possible role in selected sight-threatening disease: among 24 reported patients with DON, 88% improved in visual acuity, 75% improved after only two infusions, and mean proptosis improved by 4.37 mm, although the evidence remains observational and uncontrolled [75].

However, less selected real-world series suggest that regression and reactivation may be more common outside clinical trials. One case series saw the proptosis response rate fall from 84% to 57% at one year, with a 47% reactivation rate [76]. A single-center cohort found regression in 65.4% of improved eyes, with 25.6% ultimately becoming worse than baseline [77]. Re-treatment data remain limited: one multicenter cohort reported that 24% of patients required re-treatment at one year [78], whereas another small, non-randomized comparison reported greater proptosis improvement after a second teprotumumab course than after steroid treatment (4 mm versus 0 mm; *n* = 4 versus *n* = 6) [79]. These findings support the possible efficacy of re-treatment but do not establish superiority over alternative therapy.

Lastly, in the first placebo-controlled trial in TED of at least two years’ duration and CAS ≤ 1, teprotumumab significantly reduced proptosis (least-squares mean −2.41 mm versus −0.92 mm; *p* = 0.0004), with a 61.9% versus 25.0% responder rate [31]. This establishes the potential efficacy of IGF-1R inhibition beyond the classic active inflammatory phase, albeit with attenuated effects and without implying that all immunomodulatory therapies are effective in chronic inactive TED.

## 9. Other Targeted Biologics and the Emerging Pipeline

Beyond teprotumumab, the therapeutic landscape of thyroid eye disease is expanding along several mechanistically distinct axes (Figure 1), including the use of the interleukin-6 receptor blockade, B-cell depletion, neonatal Fc receptor inhibition, TSHR antagonism, and a widening class of insulin-like growth factor-1 receptor antagonists. These exploit the convergent immunopathology of Graves’ orbitopathy, in which IL-6 amplifies orbital fibroblast activation, autoreactive B cells generate pathogenic anti-TSH-receptor antibody (TSH-R-Ab), and IGF-1R partners functionally with the TSH receptor (Table 3). Where peer-reviewed data are unavailable, preliminary company press releases and conference reports are explicitly identified as such and are interpreted cautiously.

**IL-6R blockade.** Tocilizumab, an anti-IL-6R monoclonal antibody, is the most mature non-IGF-1R biologic. IL-6 signaling is implicated in TED pathogenesis through effects on Th17/Treg imbalance, B-cell and plasma-cell differentiation, autoantibody production, orbital fibroblast activation, vascular endothelial growth factor (VEGF)-mediated edema, and profibrotic remodeling [24]. In a randomized, placebo-controlled trial of corticosteroid-resistant moderate-to-severe disease, a CAS reduction of at least 2 points at week 16 occurred in 93.3% of treated patients versus 58.8% with placebo (odds ratio 9.8; *p* = 0.04) [25]. A 2025 single-arm meta-analysis of 219 patients corroborated this result, with a mean CAS reduction of 4.60 points, a mean proptosis reduction of 2.04 mm, and a low reactivation rate [80]. The same IL-6/IL-6R review noted reductions in thyroid-stimulating immunoglobulin titers and a relapse rate of 8.2% across reported tocilizumab studies after discontinuation [24]. The companion antibody sarilumab, studied retrospectively in low-activity disease, reduced the mean CAS from 2.22 to 0.05 (*p* < 0.001) and achieved complete response in 61.3% of patients, though adverse reactions were frequent [81].

**CD20 depletion.** Treatment of TED with rituximab has yielded inconsistent trial results. In the Salvi randomized study, the 24-week responder rate was 100% with rituximab versus 69% with intravenous methylprednisolone (*p* < 0.001), with no reactivations among rituximab patients [61]. A contemporaneous placebo-controlled trial reached the opposite conclusion, finding no benefit for active disease [82]. B-cell depletion may help only in genuinely active, earlier-stage disease; and thus, rituximab is now regarded as a second-line treatment.

**FcRn inhibition.** Batoclimab lowered total serum IgG by a dose-related 30–60% and reduced both total/binding TRAb and functional stimulatory anti-TSHR antibodies, but its phase 2 trial missed the primary proptosis endpoint and was terminated early after a dose-dependent rise in cholesterol [83]. Furthermore, both crucial phase 3 trials subsequently failed to reach the 2 mm proptosis endpoint at week 24; therefore, subsequent development has pivoted to a successor molecule [84].

**Expanding IGF-1R class.** The IGF-1R antibody IBI311 has reached a published phase 3 readout: in RESTORE-1, 85.8% of treated patients were proptosis responders at week 24 versus 3.8% with placebo (*p* < 0.001) [85]. Veligrotug (VRDN-001; veligrotug-vvze as assigned by the FDA) is no longer only a pipeline therapy: the phase 3 THRIVE trial was published in 2026 and showed a week-15 proptosis responder rate of 70% versus 5% with placebo, and the US FDA approved veligrotug for TED, regardless of activity or duration [86,87]. The half-life-extended subcutaneous successor elegrobart (VRDN-003) has reported positive company topline results in both active TED (REVEAL-1: 54% and 63% proptosis responder rates for Q4W and Q8W dosing versus 18% for placebo) and chronic TED (REVEAL-2: 50% and 54% versus 15% for placebo), but peer-reviewed publication is pending [88,89]. Another subcutaneous formulation of IGF-1R antagonism (lonigutamab) produced early responses in a phase 1/2 study [90]. The oral inhibitor linsitinib was positive in the phase 2b/3 LIDS trial, with a 52% proptosis responder rate at week 24 and hyperglycemia in only 1 of 29 patients in a small, as-yet-unpublished topline dataset for which peer-reviewed safety data are awaited [91]. The TSH-receptor-directed autoantibody K1-70 was well tolerated in a phase 1 trial, suppressing thyroid-stimulating antibody and improving proptosis in half of treated eyes [17].

Interestingly, a network meta-analysis comparing treatment effects of rituximab, tocilizumab, and teprotumumab found tocilizumab to be associated with the largest CAS reduction, while teprotumumab was associated with the largest proptosis reduction. However, the absence of randomized head-to-head trials means that such comparisons should be interpreted as hypothesis-generating rather than as definitive evidence of biologic superiority [92]. These data suggest complementary profiles, and agent selection remains individualized to phenotype, activity, severity, comorbidities, availability, and tolerability.

## 10. Surgical Rehabilitation and Sight-Threatening Disease

Surgery in thyroid eye disease serves two temporally distinct goals: rehabilitation of stable orbital, motility, and eyelid deformities once the disease has burned out, and urgent decompression to preserve vision in sight-threatening disease (Figure 4) [26].

Orbital decompression is the cornerstone procedure, indicated for dysthyroid optic neuropathy refractory to medical therapy, severe corneal exposure unresponsive to lubrication, recent globe subluxation, and—in the inactive phase—disfiguring proptosis [26]. The operation expands bony orbital volume by debulking the medial, inferior, and/or lateral walls and excising orbital fat, allowing the contents to prolapse into the adjacent sinuses or temporal fossa.

Proptosis reduction scales with the number of walls removed and the volume of fat excised. In a 2024 meta-analysis, three-wall decompression produced the greatest mean exophthalmos reduction (−5.80 mm; 95% CI, −6.47 to −5.13 mm), exceeding the results for balanced, single-wall, and fat-removal approaches [93]; two-wall decompression likewise yields substantial, durable proptosis reduction [94]. Preoperative Hertel exophthalmometry strongly predicts the achievable reduction [95].

The principal complication is new or worsened primary-gaze diplopia from asymmetric repositioning of the globe and muscles. The pooled rate was approximately 11% but was strongly technique-dependent—reaching only about 3% for lateral-wall and fat-removal approaches [93]. The deep lateral wall is therefore favored as a low-diplopia rehabilitative option, with mean proptosis reductions of roughly 3 to 4 mm [96].

When undertaken for functional and cosmetic rehabilitation, decompression is the first of a staged sequence: decompression, then strabismus surgery, followed by eyelid surgery, because each step alters the parameters addressed by the next [97]. Surgery is deferred until the disease is inactive, and the patient is euthyroid. Specifically, eyelid position should be stable for 6 to 12 months, while ocular misalignment should be stable for at least 6 months. However, urgent surgery is warranted for emergent exposure keratitis and DON [97].

DON is a sight-threatening medical emergency. DON occurs in roughly 3% to 5% of patients, with diabetes, older age, smoking, and active hyperthyroidism as risk factors [26,28,29]. Diagnosis rests on constellation of exam findings—reduced color vision, abnormal fields, a relative afferent pupillary defect, and apical crowding on imaging—because the optic disc can deceptively appear normal along with modest proptosis in a substantial minority [27,98]. First-line treatment is very-high-dose IV methylprednisolone (about 0.5–1 g on three consecutive days, repeated if response is incomplete, kept below the approximately 8 g safety ceiling), with prompt orbital decompression when vision is severely reduced at presentation or fails to improve within one to two weeks [26]. Decompression for DON has been found to yield good long-term visual recovery [99]. Small observational studies suggest that teprotumumab may reduce proptosis and improve visual function in selected DON cases, but the evidence does not establish equivalence to the results for high-dose intravenous glucocorticoids or urgent orbital decompression [75,100]. Teprotumumab use for DON remains off-label and costly; prompt steroid therapy, along with surgical decompression, retains its central role when visual compromise is severe, progresses, or fails to respond rapidly to medical therapy.

## 11. Limitations of the Clinical Activity Score and the Reproducibility Gap

The treatment decisions discussed above depend not only on CAS but also on disease severity, progression, visual function, corneal status, diplopia, and imaging findings. Nevertheless, CAS remains the dominant operational activity instrument in trials and guidelines. The initial-visit CAS is an unweighted sum of seven binary inflammatory signs, each contributing one point, with a 3/7 threshold commonly used to define active disease; at follow-up, three progression items extend the score to 10 points [30]. This construction assumes that the signs are equally informative and equally reliable.

That assumption, unfortunately, fails in practice. When the same patients are scored by multiple observers, the total CAS achieves only moderate agreement (Krippendorff alpha 0.532), and agreement on the decisive CAS ≥ 3 cut is poor; observers concurred with the lead ophthalmologist only 64.5% of the time [33]. Individual signs vary several-fold in regards to reliability: spontaneous pain is reasonably reproducible, whereas eyelid redness, chemosis, and caruncular edema are the weakest links [33,34,35]. The CAS weights them all identically.

A sum of equally weighted signs is therefore a blunt tool. It also captures only inflammation and performs weakly regarding chronic, fibrotic disease, where activity has waned but morbidity persists.

Several groups are working to improve reproducibility. Standardized photographic grading reduces some inter-observer variability but is itself only fairly to moderately reliable [34]. Machine-learning scoring from facial images offers a more objective, automated read-out [38], and quantitative orbital MRI markers promise an observer-independent activity signal.

None of these has displaced the CAS. Developing—and, critically, prospectively validating—a more reliable, more reproducible activity measure remains an open priority for the field. The needed evidence is a prospective cohort with paired multi-observer grading, objective markers, and treatment-response outcomes.

## 12. Conclusions and Future Directions

Thyroid eye disease management and understanding have evolved more significantly over the past five years than in the preceding fifty (Figure 5). It is now a treatable orbitopathy whose biology, centered on the orbital fibroblast and its TSHR–IGF-1R signaling, has yielded validated targets and, for the first time, agents that reverse proptosis. The frontier has shifted from whether the orbit can be modified pharmacologically to how to do so precisely, durably, and affordably.

The Clinical Activity Score remains the most widely used operational definition of active disease, yet it should not be treated as the sole gatekeeper for therapy. Treatment decisions also require the consideration of severity assessment, disease progression, visual function, corneal status, diplopia, imaging findings, comorbidities, and patient goals. Its inter-observer reliability is only moderate, and its decisive treat/no-treat threshold shows poor inter-observer agreement (concordance of about 65%) [33]. A prospective, multi-observer cohort linking activity scores, objective biomarkers, and treatment-response outcomes would put the threshold that guides therapy on firmer footing and help identify more reproducible measures for tracking disease activity and therapeutic response.

Currently, teprotumumab and the broader IGF-1R antagonism class have produced the largest medically achieved proptosis reductions, and veligrotug has now expanded the approved US IGF-1R treatment class. However, the absence of randomized head-to-head trials against intravenous glucocorticoids, otologic and metabolic toxicity, rebound after discontinuation, high per-course cost, and evolving access constraints leave optimal sequencing uncertain at this time. Trials reporting rebound rates, re-treatment efficacy, and outcomes in chronic, low-activity disease are needed to convert this expanding pipeline into rational practice.

Euthyroidism, smoking cessation, and ocular-surface protection are foundational; glucocorticoids and biologics address active inflammation; decompression, strabismus realignment, and eyelid surgery rehabilitate the stable orbit. Dysthyroid optic neuropathy remains an emergency. Reliable activity measurement is the prerequisite for the personalized, evidence-based care this disease now permits.

## Figures and Tables

**Figure 1:**
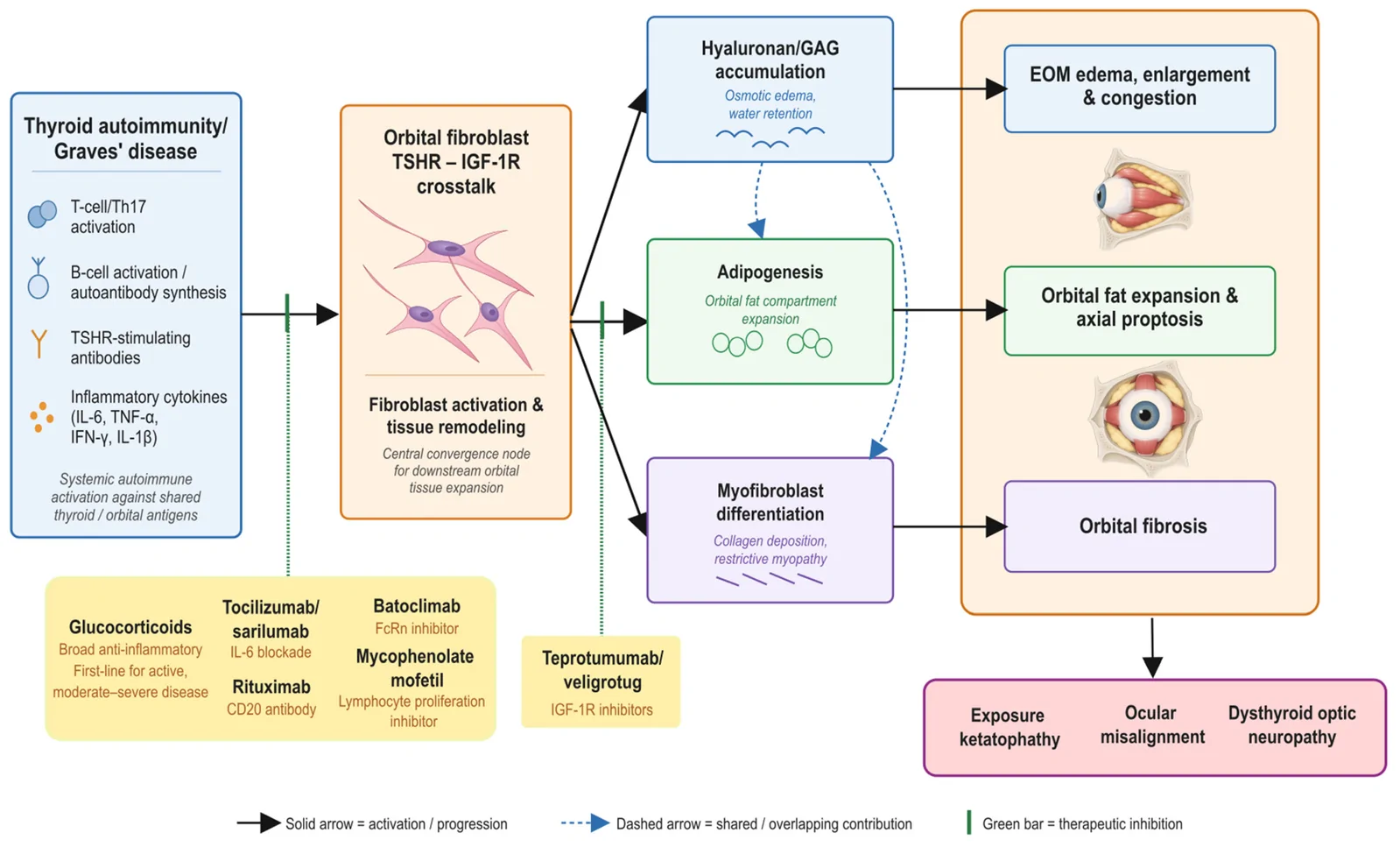
Pathophysiology of thyroid eye disease mapped to therapeutic targets. Thyroid autoimmunity activates the orbital fibroblast through TSHR–IGF-1R crosstalk. Fibroblast activation then drives overlapping hyaluronan/glycosaminoglycan (GAG) accumulation, adipogenesis, and myofibroblast differentiation. Increased hyaluronan/GAG production is shown as a shared contributor to both adipogenic and myogenic tissue expansion rather than as an isolated pathway. These processes produce extraocular muscle (EOM) edema and enlargement, orbital fat expansion with axial proptosis, and progressive orbital fibrosis with restrictive strabismus and diplopia. Posterior EOM enlargement and apical crowding may culminate in dysthyroid optic neuropathy, while proptosis and eyelid malposition contribute to ocular-surface exposure. Corresponding drug classes are mapped onto their molecular targets.

**Figure 2:**
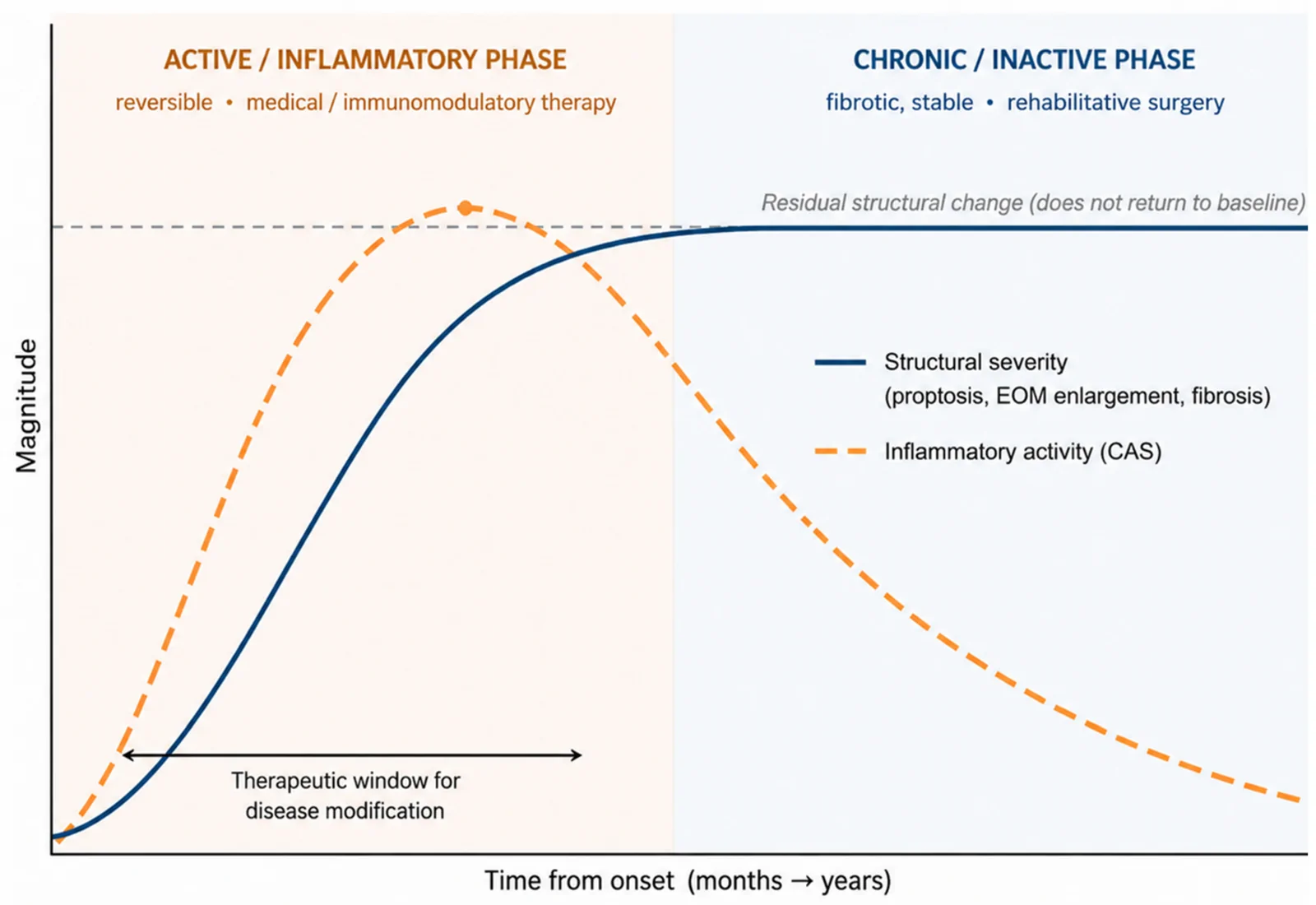
Rundle’s curve illustrating the natural history of thyroid eye disease over time from disease onset (months to years). Inflammatory activity rises and subsequently resolves, representing the principal reversible window for medical therapy, whereas structural severity increases and may plateau at a residual level that persists into the chronic inactive phase and is traditionally managed with rehabilitative surgery.

**Figure 3:**
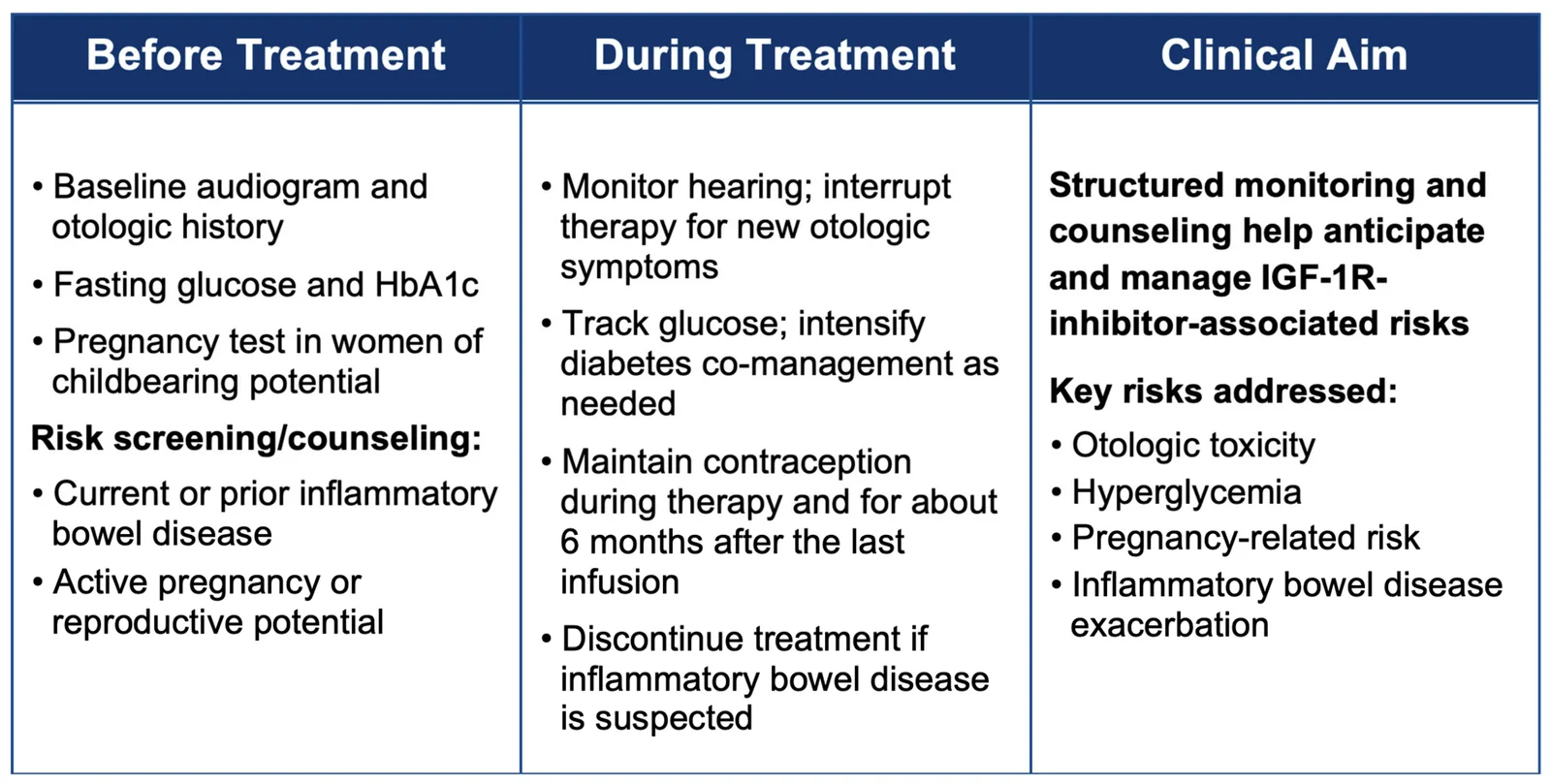
Teprotumumab monitoring and counseling protocol. Pretreatment screening should assess otologic history and audiogram, glycemic status, pregnancy risk, and inflammatory bowel disease (IBD) history and symptoms. During therapy, structured monitoring of hearing, glucose control, contraception, and gastrointestinal symptoms may help anticipate and manage treatment-associated risks.

**Figure 4:**
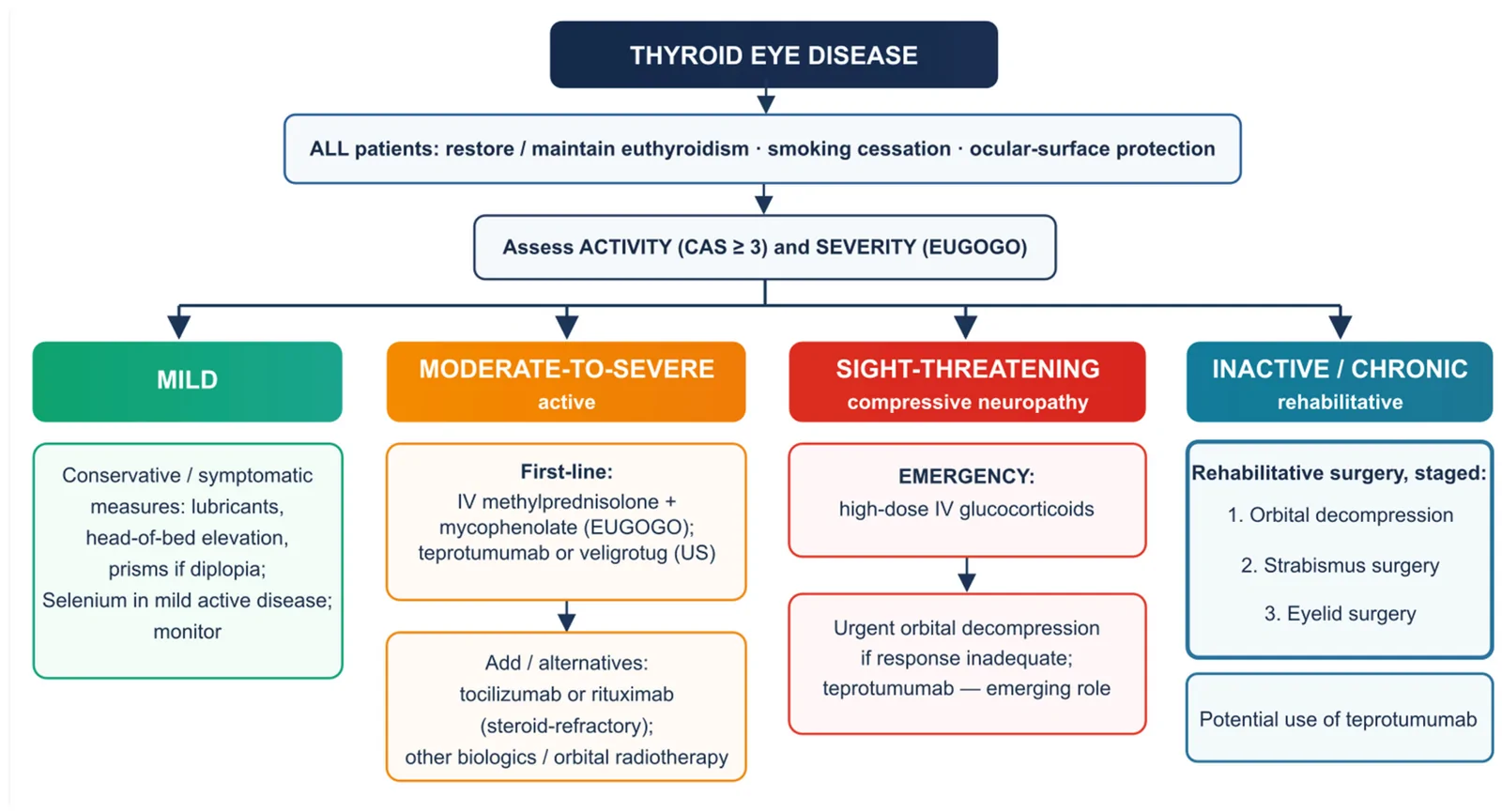
Management of thyroid eye disease according to disease activity and severity. Baseline measures apply to all patients; treatment then follows the activity/severity category. Timing and sequence depend on disease activity and euthyroid status. Categories may overlap as care is individualized.

**Figure 5:**
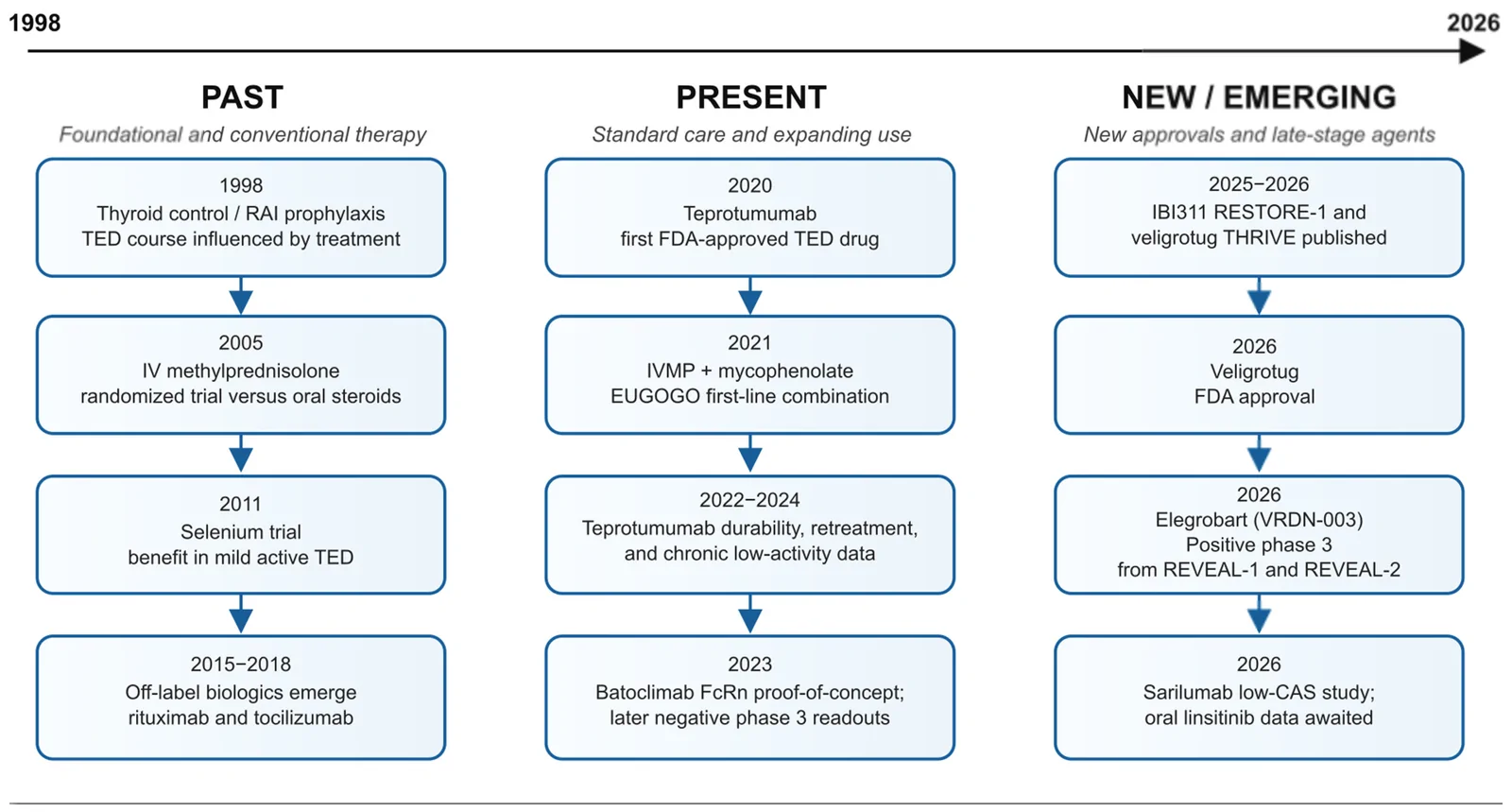
Timeline of advances in thyroid eye disease treatment. Dates represent treatment milestones rather than definitive first historical use. The timeline moves from supportive care and broad immunosuppression to off-label biologics, FDA-approved teprotumumab, newly approved veligrotug, chronic-disease and retreatment data, and the emerging IGF-1R, FcRn, IL-6R, CD20, and TSHR-directed therapeutic pipeline employing different delivery routes/formulations.

**Table 1:** The ten-item Clinical Activity Score (CAS) [30]. Only the first seven items are assessed at initial visit. One point is scored for each item present; a sum of ≥3/7 at the initial visit defines active disease. At follow-up visits, three additional items (increase in proptosis ≥ 2 mm, decrease in eye movement ≥ 8 degrees, decrease in visual acuity ≥ 1 Snellen line over 1–3 months) are evaluated, extending the scale to 10 points. Inter-observer reliability is only moderate [33].

Item No.	Clinical Activity Score Item
1	Spontaneous retrobulbar pain
2	Pain on attempted upgaze or downgaze
3	Eyelid erythema
4	Conjunctival injection
5	Chemosis
6	Swelling of the caruncle or plica
7	Eyelid edema or swelling
8	Increase in proptosis ≥ 2 mm
9	Decrease in eye movements ≥ 8 degrees
10	Decrease in visual acuity ≥ 1 Snellen line over 1–3 months

**Table 2:** Complementary roles of CT, MRI, and 99mTc-DTPA SPECT/CT in thyroid eye disease. CT and MRI remain the principal cross-sectional modalities; 99mTc-DTPA SPECT/CT is an adjunctive functional technique for activity assessment in selected centers and remains less standardized than CT or MRI [41,42,43,44,45,46,47,48,49,50,51].

Feature	CT	MRI	99mTc-DTPA SPECT/CT
**Bony orbit/surgical anatomy**	Excellent; modality of choice for decompression planning	Limited	Limited anatomical detail; fused CT helps localization
**Soft tissue/optic nerve**	Adequate	Superior soft-tissue and optic-nerve resolution	Functional uptake reflects inflammatory activity more
**Ionizing radiation**	Yes	None	Yes
**Activity quantification**	No; reflects accumulated volume	T2 SIR; relaxometry; DWI/ADC; dynamic enhancement	Orbital/EOM uptake ratios and SUVmax; correlate with clinical activity
**DON/apical crowding**	Apical crowding and Barrett index	Optic nerve and apical soft-tissue characterization	Investigational adjunct; not first-line for emergency
**Speed, cost, availability**	Fast; inexpensive; widely available	Slower; costlier; less accessible	Expensive; much less accessible; needing nuclear-medicine availability
**Best use**	Surgical planning; rapid apex assessment	Activity staging; serial monitoring; radiation avoidance	Adjunctive activity assessment/research where available

**Table 3:** Pharmacotherapy for moderate-to-severe and sight-threatening thyroid eye disease. Numbers are representative findings and include a mixture of peer-reviewed trial data and explicitly labeled preliminary company topline data; treatment choice depends on activity, severity, comorbidities, availability, regulatory status, and patient preference.

Agent	Target	Representative Evidence	Key Effect	Notable Safety
IV methylprednisolone	Broad immunosuppression	EUGOGO first-line; 77% vs. 51% oral	Inactivates about 45–65%; relapse about 31%	Hepatic and cardiovascular risk; avoid cumulative doses >8 g
Mycophenolate	Lymphocyte proliferation	MINGO: benefit significant at week 24; EUGOGO adjunct	Improves response and sustained response	GI intolerance; teratogenic
Orbital radiotherapy	Local anti-inflammatory	Adjunct to glucocorticoids in selected active disease	May improve motility/activity	Caution in diabetic or hypertensive retinopathy
Tocilizumab	IL-6R	RCT: CAS response 93% vs. 59%	Reduces activity in steroid-resistant disease	Infection risk; lipid changes
Rituximab	CD20-positive B cells	Discordant RCTs	Possible benefit in early active disease; second line	Infusion reactions
Teprotumumab	IGF-1R	OPTIC: 83% vs. 10% proptosis response	Proptosis −2.8 mm; diplopia improvement	Otologic toxicity; hyperglycemia; IBD warning; fetal-harm risk
Veligrotug	IGF-1R	THRIVE published; FDA-approved in 2026	70% vs. 5% proptosis response at week 15; 5 IV infusions	Class otologic and hyperglycemic risk; IBD warning
IBI311; lonigutamab; elegrobart	IGF-1R	RESTORE-1; lonigutamab early data; REVEAL topline	Expanding class of IGF-1R antagonism and delivery routes	Class safety; peer-reviewed data incomplete for some agents
Linsitinib, oral	IGF-1R	Phase 2b/3 LIDS: topline 52% response	Oral dosing	Peer-reviewed efficacy/safety data awaited
Batoclimab	FcRn	Phase 3 failed proptosis endpoint	No significant proptosis benefit	Dose-dependent lipid rise

## Data Availability

No new data were created or analyzed in this study. Data sharing is not applicable to this article.
